# Greater Loss of Central Adiposity from Low-Carbohydrate versus Low-Fat Diet in Middle-Aged Adults with Overweight and Obesity

**DOI:** 10.3390/nu13020475

**Published:** 2021-01-31

**Authors:** Valene Garr Barry, Mariah Stewart, Taraneh Soleymani, Renee A. Desmond, Amy M. Goss, Barbara A. Gower

**Affiliations:** 1 Department of Nutrition Sciences, School of Health Professions, The University of Alabama at Birmingham, 1720 University Blvd, Birmingham, AL 35294, USA; amc3321@uabmc.edu (M.S.); soltar@uab.edu (T.S.); amymiski@uab.edu (A.M.G.); bgower@uab.edu (B.A.G.); 2 Division of Preventive Medicine, The University of Alabama at Birmingham, Medical Towers 621, 1717 11th Avenue South, Birmingham, AL 35205, USA; rdesmond@uabmc.edu

**Keywords:** weight loss, low-carbohydrate diet, insulin resistance, visceral fat, DXA

## Abstract

The objective of this study is to determine whether middle-aged adults prescribed a low carbohydrate-high fat (LCHF) or low fat (LF) diet would have greater loss of central fat and to determine whether the insulin resistance (IR) affects intervention response. A total of 50 participants (52.3 ± 10.7 years old; 36.6 ± 7.4 kg/m2 BMI; 82% female) were prescribed either a LCHF diet (*n* = 32, carbohydrate: protein: fat of 5%:30%:65% without calorie restriction), or LF diet (*n* = 18, 63%:13–23%: 10–25% with calorie restriction of total energy expenditure—500 kcal) for 15 weeks. Central and regional body composition changes from dual-x-ray absorptiometry and serum measures were compared using paired *t*-tests and ANCOVA with paired contrasts. IR was defined as homeostatic model assessment (HOMA-IR) > 2.6. Compared to the LF group, the LCHF group lost more android (15.6 ± 11.2% vs. 8.3 ± 8.1%, *p* < 0.01) and visceral fat (18.5 ± 22.2% vs. 5.1 ± 15.8%, *p* < 0.05). Those with IR lost more android and visceral fat on the LCHF verses LF group (*p* < 0.05). Therefore, the clinical prescription to a LCHF diet may be an optimal strategy to reduce disease risk in middle-aged adults, particularly those with IR.

## 1. Introduction

Overweight and obesity substantially increase the risk of adverse health events for middle-aged and older adults, such as heart attacks, strokes, and diabetes [1]. The growing obesity epidemic has increasingly led healthcare providers to prescribe dietary interventions to mitigate such poor outcomes. However, choosing the best dietary intervention for those with advanced age remains controversial [2]. An optimal weight loss intervention to reduce disease risk in these populations should target central fat depots, which are pathogenic and grow at a higher rate relative to total body fat in people with advanced age [3]. Additionally, interventions should consider age-specific health concerns, such as the preservation of lean mass and improvement of dyslipidemia and dysglycemia to prevent sarcopenia and reduce disease risk, respectively [2,4]. Yet, there is limited evidence that indicates how particular diet interventions affect these age-specific targets among middle-aged to older adults [2,5].

A low carbohydrate-high fat (LCHF) diet may be ideal for weight loss in an older population. LCHF diets restrict carbohydrate consumption to < 45% of energy intake and emphasize limits on starch and sugar, while encouraging the consumption of healthy (mono- and polyunsaturated) fats and the maintenance of moderate protein intake [6,7]. LCHF diets are known for rapid weight loss with a greater reduction in body fat and preservation of lean mass, compared to diets that are lower in fat [7,8,9]. Further, LCHF diets have been associated with a preferential loss of visceral and ectopic fat among younger adults and limited evidence supports similar benefits in older adults [10]. Such results would be ideal for older adults and would mitigate concerns about sarcopenia while targeting the most metabolically harmful fat depots. However, the existing evidence is insufficient to determine whether this diet response is consistent across age groups [2,10].

LCHF diets may also be ideal for individuals with insulin resistance, a condition that is common among older adults and is associated with higher cardiometabolic disease risk [11,12]. Insulin resistance occurs in concert with compensatory hyperinsulinemia, which may prevent mobilization of fatty acids when weight loss is attempted. The lower carbohydrate content of the LCHF diet is believed to reduce insulin secretion, which may permit greater lipolysis and fat oxidation during negative energy balance [7]. In support of this hypothesis, studies that have stratified participants by either insulin resistance or fasting insulin have showed that diet intervention outcomes differ according to phenotype, and that those with insulin resistance responded better to low-carbohydrate diets [13,14,15]. However, similar studies have not been conducted in middle-aged to older adults [2,16]. 

The primary objective of this study was to examine whether changes in regional fat and lean mass (kg) differed between middle-aged and older adults with overweight and obesity that were prescribed either a LCHF or LF diet for 15 weeks. Our second objective was to determine whether insulin resistance status affected changes in body composition following the intervention period. We hypothesized that a LCHF diet would result in greater loss of total and central fat mass with lower loss of lean mass, compared to LF, and that this response would be particularly pronounced in individuals with insulin resistance.

## 2. Materials and Methods

### 2.1. Participants and Recruitment

Between May 2014 and January 2017, participants were recruited from a medically supervised weight loss clinic at The University of Alabama at Birmingham (UAB). Additionally, participants were recruited by flyer, newspaper, web-based advertisement, and word of mouth. Eligible participants had a body mass index (BMI) ≥ 25 kg/m^2^, had been weight stable (<10 pounds gained or lost) in the prior 12 months, and were not actively engaged in an exercise program. Participants with type 2 diabetes were eligible if they were not insulin dependent. Exclusion criteria included age > 75 years, BMI > 50 kg/m^2^, current smoking, pregnancy, or breastfeeding. Eligibility was determined during a telephone screening session and confirmed during an in-person screening visit, where participants were also informed of the study procedures and provided both verbal and written consent. The UAB Institutional Review Board (IRB) approved this study.

The goal of these analyses was to examine the effects of diet prescriptions on middle-aged to older (45–75 years) adults. To detect a visceral fat loss of 11.1 ± 8.8% with a two-sided 5% significance level and a power of 80%, a sample of 24 participants per group were necessary. We anticipated a dropout rate of 25%.

Some participants younger than 45 were recruited. Given the target age range, we conducted analyses both with and without participants younger than 45 (*n* = 7; 5 LCHF, 2 LF). We observed no differences in study outcomes when younger participants were omitted. Thus, all participants who completed baseline and follow-up assessments were included in the analysis. Participants with missing outcome data were omitted [17].

### 2.2. Diet Prescriptions

#### 2.2.1. Low-Carbohydrate High-Fat Diet (LCHF)

The LCHF prescription had a target macronutrient ratio (carbohydrate: protein: fat) of 5%:30%:65% of total energy, and encouraged increased monounsaturated and polyunsaturated fat intake. At initiation, participants were instructed to consume three meals per day with up to two snacks per day. Daily carbohydrate was limited to no more than 20 total grams per day with 12–15 g from vegetables. The LCHF guidelines and corresponding meal plans recommended four ounces of protein with each meal, four ounces of dairy per day, one cup cooked non-starchy vegetables daily, two cups leafy vegetables daily, and additional energy needs should come from healthy fats such as ½ an avocado, olives, and olive oil. After eight weeks with the LCHF prescription, participants were allowed to increase carbohydrate intake to 30 g per day and were allowed to add nuts, nut butters, and berries into their diets.

#### 2.2.2. Low-Calorie Low-Fat Diet (LF)

The LF diet prescription had a target a macronutrient ratio (carbohydrate: protein: fat) of 63%:13–23%:10–25% of total energy and restricted calories to 1200–1600 kcal per day based on a 500 kcal reduction from baseline resting energy expenditure (REE) *1.3 [18]. The LF diet emphasized the consumption of low energy density foods, appropriate portion control, and specified low fat food options to meet energy needs. The LF guidelines and corresponding meal plans recommend that where possible, participants chose vegetables over fruit, and fruit over starches. Likewise, guides recommend that participants consume dairy more often than meat or protein sources, and meat or protein more often than fat.

#### 2.2.3. Diet Selection and Adherence

After reviewing guidelines for both diets with a registered dietitian (RD), participants consulted with a weight loss medicine physician to choose their diet prescription. Then an RD provided diet instructions, sample meal plans, and recipes to aid participants with adhering to their respective diet. In addition, participants were offered weekly group lifestyle management classes that provided accountability and discussed topics such as Quick Cooking and Meal Assembly, Mindful Eating, and Healthy Substitutions on LCHF or LF. Diet adherence was monitored via 3-day food records and body weight by the study physician and RD at dedicated follow-up sessions that occurred at +2 weeks, +4 weeks, +8 weeks, +12 weeks, and +15 weeks.

### 2.3. Study Outcomes and Tests

Study outcomes were assessed before (within one week) diet initiation and after 15 weeks of the intervention, which included standard anthropometrics, fasting serum measures, REE, and body composition. All measurements and tests were completed in the Core facilities of the Center for Clinical and Translational Science (CCTS), Nutrition Obesity Research Center (NORC), and Diabetes Research Center (DRC) at UAB. Participants were instructed to fast for at least 10 h (overnight) before each study visit, maintain their usual activity level, to avoid strenuous exercise the day before testing, and avoid exercise on the morning of testing.

#### 2.3.1. Body Composition

Total and regional fat and lean mass were assessed using dual X-ray absorptiometry (DXA) (Lunar iDXA, enCORE version 13.6, GE Medical Systems, Madison, WI, USA) [19,20,21] using manufacturer defined regions of interest (ROI), which are described in detail elsewhere [21,22]. Fat and lean mass were analyzed for each of the following regions: (1) total body, which includes all regions in the scan; (2) trunk, which includes neck, chest, abdominal, and pelvic areas; (3) android, which includes the area between ribs and pelvis (totally enclosed inside the trunk); (4) gynoid, which includes hips and upper thighs, and overlaps with both legs and trunk; and (5) appendicular, which includes arms (and shoulder) and leg regions [19,21]. Visceral fat was estimated using the CoreScan algorithm (GE Medical Systems, Madison, WI, USA), which subtracts subcutaneous fat from total android fat to yield an estimate of visceral fat [23].

#### 2.3.2. Resting Energy Expenditure

REE and respiratory quotient (RQ) were measured using a VMAX ENCORE 29N indirect calorimeter [VIASYS Respiratory Care, Inc. (formerly Sensormedics), Palm Springs, CA] following an overnight fast and a 15-min supine rest.

#### 2.3.3. Glucose, Insulin and Lipids

Fasting blood draws were performed at each study visit to measure glucose, insulin, and lipids. Glucose and lipids were analyzed using the SIRRUS analyzer (Stanbio Laboratory, Boerne, TX, USA). Glucose was measured in 3μL sera using the glucose oxidase method. This analysis had an intra-assay coefficient of variation (CV) of 1.2% and inter-assay CV of 3.1%. Insulin was assayed in 50 μL aliquots using immunofluorescence technology on a TOSOH Automated Immunoassay Analyzer II (AIA-II) analyzer (TOSOH Corp., South San Francisco, CA, USA). This analysis had an intra-assay CV of 1.5% and inter-assay CV of 4.4%. Insulin and glucose were used to quantify insulin resistance according to the homeostatic model assessment of insulin resistance (HOMA-IR) [24]. Insulin resistance was defined as HOMA-IR > 2.6 [25]. Baseline glycated hemoglobin (HbA1C) > 6.5% was used to denote diabetes [26].

### 2.4. Statistical Analysis

Baseline descriptive statistics (means ± SD) were compared between groups using the Student’s *t*-tests for continuous variables and the Fisher’s exact test for categorical variables. Serum measures that were not normally distributed were log-transformed for analyses. Change values (follow-up–baseline) for regional fat mass, lean mass, and serum measures were evaluated for by paired *t*-tests and compared between-groups using analysis of covariance (ANCOVA) controlling for the respective baseline measure, age (years), sex, and race and ethnicity. Due to our small sample size, this study was not powered to test for interactions of insulin resistance status with every outcome. Therefore, the effects of insulin resistance phenotype on the response to the intervention were explored with subgroup analysis via pairwise contrasts. Statistical significance was specified as *p* ≤ 0.05.

## 3. Results

### 3.1. Baseline Characteristics

In total, 80 participants enrolled in the study and chose either the LCHF (*n* = 48) or LF (*n* = 32) diet. The overall study completion rate was 63% (*n* = 50) and was not statistically different between groups; 67% (*n* = 32) for the LCHF and 56% (*n* = 18) for the LF group. The baseline characteristics were not significantly different between completers and non-completers.

The participants (henceforth, meaning those who completed the study) were 82% (*n* = 41) women and 74% (*n* = 37) European Americans with a mean age of 52 ± 10 years. At baseline, 52% (*n* = 26) of participants were insulin-resistant (HOMA-IR > 2.6) and 14% (*n* = 7) had diabetes (HbA1c > 6.5). The participants’ baseline characteristics were not significantly different between the LCHF and LF groups (Table 1). Self-reported medication use and medical history are shown in the (supplementary materials Tables S1 and S2).

### 3.2. Total Fat and Lean Mass Loss

**Figure 1:**
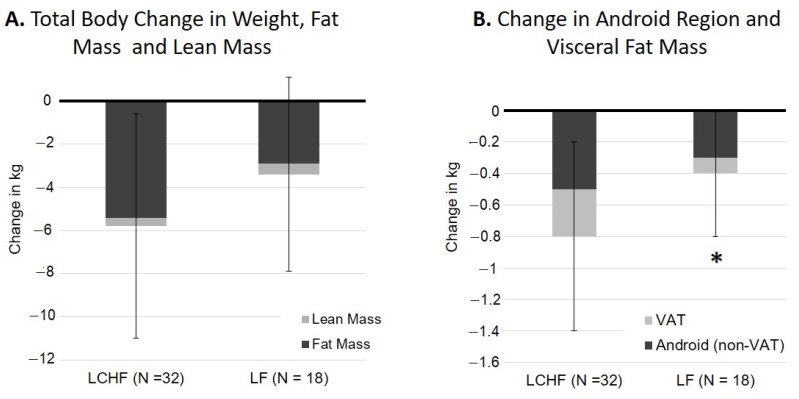
Age, sex, and race, adjusted comparison of change in body composition by diet group. (**A**) Change in weight (total bar), fat mass (dark-filled segment) and lean mass (light-filled segment). (**B**). Change in android region fat mass (total bar), non-visceral android fat (dark-filled segment) and visceral fat (VAT) mass (light-filled segment). Compared to the Low-carbohydrate high fat (LCHF) group, the low-fat (LF) had lower changes in android regions and visceral fat. * (*p* < 0.05).

At follow-up, participants in both groups had a significant decrease in total body weight and total body fat mass, but maintained total body lean mass (Table 2). The LCHF group lost 6.1 ± 5.2 kg (5.4 ± 3.6 kg fat), and the LF group lost 3.1 ± 4.5 kg (3.0 ± 3.2 kg fat). There were no significant differences in weight, total fat, or total lean mass changes between diet groups (Figure 1A).

### 3.3. Regional Fat and Lean Mass Loss

Both groups had a significant decrease in trunk, android, and gynoid fat mass (Table 2). The LCHF group had a significant decrease in visceral fat (*p* < 0.001). The LF group did not have any significant change in visceral fat (*p* = 0.40). Compared to the LF group, the LCHF group had significantly greater loss of visceral fat (18.5 ± 22.2% vs. 5.1 ± 15.8%, *p* < 0.05) and total android region fat (15.6 ± 11.2% vs. 8.3 ± 8.1%, *p* < 0.01) (Figure 1B). However, there were no significant differences in loss of gynoid or appendicular fat mass between the groups. With respect to lean mass, the LCHF group lost lean mass in the trunk (*p* < 0.05), android (*p* < 0.01), and gynoid (*p* < 0.01) regions, but maintained total body and appendicular lean mass. In contrast, the LF group did lose a significant amount of lean mass in any region (Table 2).

### 3.4. Serum Measures

In the LCHF group, HOMA-IR significantly decreased (*p* < 0.001). Conversely, in the LF group, HOMA-IR significantly increased (*p* < 0.05) (Table 2). Although potentially clinically relevant, the difference between groups for the change of HOMA-IR (LCHF: - 0.8 HOMA units vs. LF + 0.9 HOMA units) did not quite reach statistical significance (*p* = 0.06). All other serum measures were similar to their baseline values in both groups and were not significantly different between groups (Table 2).

### 3.5. Subgroup Analysis by Insulin Resistance Status

**Figure 2:**
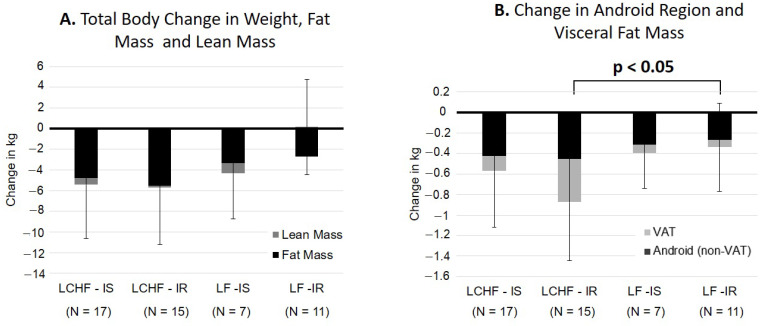
Age, sex, and race, adjusted comparison of change in body composition by diet group and insulin resistance phenotype. (**A**) Change in weight (total bar), fat mass (dark-filled segment), and lean mass (light-filled segment). (**B**). Change in android region fat mass (total bar), non-visceral android fat (dark filled segment) and visceral fat mass (light-filled segment). Changes in android regions and visceral fat were lower among insulin-resistant participants in the in the LF group versus the LCHF group (*p* < 0.05).

Between-group contrasts showed that insulin-resistant participants in the LCHF group lost significantly more android and visceral fat than insulin-resistant participants in the LF group (*p* < 0.05) (Figure 2B). Although not significant, insulin-resistant participants in the LF group gained visceral fat. Insulin-resistant individuals in the LCHF diet had a greater decrease in fasting insulin and HOMA-IR, compared to the LF group (*p* < 0.05). Conversely, there were no differences for changes in central fat mass, lean mass, or serum measures between insulin-sensitive participants that were prescribed to different diets (Table 3). Table 3 provides change values for each phenotype-diet group and *p*-values for each comparison.

Within-group contrasts showed that insulin-sensitive and insulin-resistant participants lost similar amounts of total body and central fat mass and lean mass, regardless of diet (Figure 2A). Insulin-resistant participants on the LCHF diet had a greater increase in high density lipoprotein (HDL) than insulin-sensitive participants on the same diet (*p* < 0.05) (Table 3).

## 4. Discussion

The goal of this study was to determine whether prescribing a low carbohydrate high fat diet or a low fat low-calorie diet would result in greater loss of total body and central fat mass, less loss of lean mass among middle-aged adults with overweight and obesity. We also aimed to determine whether insulin resistance status would affect response to the intervention.

The main finding of this study was that compared to the LF diets, the LCHF diet prescription elicited a greater reduction in central fat mass. After the 15-week intervention period, we observed a greater reduction in android and visceral fat in the LCHF group, even though both groups lost similar amounts of weight and total body fat mass. These findings extend previous observations from our group, which showed that carbohydrate restriction yields greater loss of intra-abdominal fat among adults with overweight or obesity and women with polycystic ovary syndrome (PCOS) [7,9]. Together, these data suggest that the LCHF diet facilitates mobilization of fatty acids specifically from central and visceral depots, which may alleviate risk for age-related cardiometabolic disease in middle-aged adults [27].

The mechanism for the preferential loss of central fat with the LCHF diet is not fully understood. However, the results of this study align with the theory that fatty acids from the visceral depot have a higher turnover rate, and are generally mobilized first during negative energy balance, which may be amplified by the reduction in insulin secretion with a LCHF diet [28]. Further, lower insulin secretion would permit greater oxidation of fatty acids as a fuel, and reduce stimulation of triglyceride uptake in adipose tissue (re-esterification) [29]. In contrast, LF (high carbohydrate) diets retain greater insulin secretion during negative energy balance, and in some studies result in loss of greater lean body mass [30]. It is possible that the higher insulin with the LF diet results in greater use of glucose and amino acids for fuel, resulting in greater re-esterification of fatty acids into adipose tissue triglyceride stores. “The higher glucagon to insulin ratio observed with reduced carbohydrate diets [31] may also play a role in redistribution of energy from central fat depots [32].

We found that total body and appendicular lean mass were preserved in both groups. However, the LCHF group lost a small but statistically significant amount of central lean mass. In this population, weight loss prescriptions should avoid excess lean mass loss due to increased risk for sarcopenia and sarcopenic obesity, which are defined by excessive loss of total body or appendicular mass [33]. Since both total and appendicular lean mass were preserved in both groups, these findings suggest that there is no greater risk associated with lean mass loss from a short-term intervention on either a LF or LCHF diet.

Multiple studies have shown that serum measures that are associated with metabolic risk markedly improve with weight loss regardless of diet [5,34,35,36,37,38]. In this study, although both groups lost weight, those prescribed to the LCHF diet had a significant improvement in insulin resistance, whereas those prescribed to the LF diet showed worse insulin resistance. Likewise, the LCHF group showed a significant decrease in triglycerides, whereas the LF had a slight and non-significant decrease. Although the difference between groups did not reach statistical significance for the change in HOMA-IR or triglycerides, these differences may be nonetheless clinically significant in individual patients.

Similar findings were reported in a recent meta-analysis of 23 randomized controlled trials that compared LCHF and LF diets. Hu et al. reported that LCHF diets result in a greater improvements in overall lipid metabolism measured by triglycerides, HDL, low-density lipoprotein (LDL), and total cholesterol [39]. Such improvements with carbohydrate restriction may result from a shift of intrahepatic metabolism away from insulin-stimulated triglyceride synthesis toward ketone body production [40]. Conversely, LF diets that are high in carbohydrates are well known to increase cholesterol and endogenous triglyceride production [41]. Hence, independent of weight loss, prescribing a LCHF diet may confer additional reduction in disease risk over a LF diet.

We found that changes in body composition were likely impacted by insulin resistance status. Insulin-resistant participants in the LCHF group lost more android, gynoid, and visceral fat and had greater improvements in HOMA-IR than those in the LF group. There was no difference in the effect of diet prescription for insulin-sensitive adults. Our results are consistent with previous evidence, showing that the insulin-resistant phenotype has a better response to low carbohydrate diets [42]. Hjorth et al. and Pittas et al., stratified their sample by insulin sensitivity phenotype and fasting insulin, respectively, and found that total body weight loss differed according to phenotype [13,15]. This study adds that those with insulin resistance lose more central fat in response to a low carbohydrate diet than those on a low fat diet. These data suggest that prescribing a LCHF diet may confer additional advantage over a LF diet for middle-aged adults with insulin resistance.

A limitation of this study was that it used a diet choice model, rather than a randomized control model. It is well established that choosing a low-carbohydrate diet is more common among individuals with dietary habits and preferences that are more consistent with the LCHF diet. However, studies show that choosing one’s diet does not influence weight loss, health outcomes, or adherence [43,44]. Second, the dropout rate in this study was 37%, which is within the normal range for diet interventions [45]. However, sensitivity analyses confirmed that bias was not introduced by attrition. It should be noted that factors such as genetics, and other variables not assessed, may have contributed to variation in the outcomes measured. Additionally, energy intake was not reported in this study. Therefore, the possibility that differences in calorie intake between the groups cannot be ruled out as a potential contributor to the observed changes in body composition. Lastly, this study had a small sample that was 82% women and 81% European American. Therefore, the results may not accurately reflect diet response among men or African Americans. The influence of sex and insulin resistance status on the response to a LCHF diet intervention should be evaluated in future studies. Nevertheless, the strengths of this study were its rigorous medical supervision and investigation of the diet-phenotype interactions. This study was conducted in a weight loss clinic, supporting the feasibility of achieving clinically meaningful improvements in metabolic health with a LCHF intervention in a clinic setting.

In summary, among middle-aged adults with overweightness and obesity, choosing a low carbohydrate-high fat diet prescription was associated with a greater central fat loss than the low-fat low-calorie diet, even though weight and total fat mass loss were comparable. Those with an insulin-resistant phenotype fared better on the low-carbohydrate-high-fat diet versus the lower fat diet for central fat loss, suggesting that evaluating patients for insulin resistance status at the time of presentation may be useful for determining the optimal diet prescription to reduce central adiposity in this population. Conversely, for those with an insulin-sensitive phenotype, diet choice did not affect outcomes, indicating that insulin sensitive patients may benefit from range of diet options. These observations suggest that LCHF diets may be a better weight loss approach for middle-aged adults with overweight and obesity, especially those with diminished insulin sensitivity.

## Figures and Tables

**Table 1:** Baseline Characteristics of Participants by Intervention (*n* = 50).

	LCHF Diet (*n* = 32)	LF Diet (*n* = 18)	*p*-Value
Age, years	52.8 (2.1)	48.4 (10.3)	0.69
BMI, kg/m^2^	36.3 (7.6)	37.4 (6.9)	0.61
REE, kcal/day	1562.6 (275.8)	1613.7 (339.6)	0.33
Gender, %			
Female	81.3	83.3	0.85
Male	18.8	16.7	
Race, %			
European American	81.3	61.1	0.12
African American	18.8	38.9	
Insulin Resistance, %			
HOMA-IR < 2.6 (IS)	70.8	57.7	0.39
HOMA-IR ≥ 2.6 (IR)	29.1	42.3	
Diabetes, %			
HbA1C > 6.5	87.5	83.33	0.69
HbA1C ≤ 6.5	12.5	16.37	
Regular Exercise, %			
Yes	42.9	31.3	0.53
No	57.1	68.8	
Dropouts, %	31.9	43.8	0.28

Data given as mean (SD) or percentage; IS—Insulin-sensitive; IR—Insulin-resistant.

**Table 2:** Comparison of Baseline and Follow-Up (15 week) Measures by Paired *t*-test Within Each Diet Group and ANCOVA Between Diet Groups.

	LCHF Diet (*n* = 32)		LF Diet (*n* = 18)		*p*-Value
	Baseline	Follow-Up	Baseline	Follow-Up	
**Demographics**					
Weight, kg	100.1 (24.7)	94.0 (24.1) ***	105.3 (18.9)	102.1 (19.4) **	0.1615
BMI, kg/m^2^	36.1 (7.7)	33.9 (7.4) ***	37.4 (6.9)	36.3 (7.1) **	0.1713
Fat, %	45.3 (7.4)	42.6 (7.7) ***	45.8 (5.2)	44.3 (5.3) ***	0.0864
REE, kcal/day	1562.6 (275.8)	1531 (285.7)	1613.7 (339.6)	1556.3 (304.9)	0.1199
**Region Fat Mass—DXA**					
Total Fat, kg	46.1 (15.8)	40.7(15.0) ***	48.7 (13.2)	45.8 (12.9) **	0.055
Appendicular Fat, kg	20.3 (8.7)	23.6 (6.4) ***	21.7 (6.8)	20.6 (7.1) **	0.193
Trunk Fat, kg	25.3 (9.1)	21.9 (8.1) ***	26.0(7.7)	24.2 (7.2) **	**0.049**
Android Fat, kg	4.6 (1.8)	3.9 (1.6) ***	4.7 (1.4)	4.3 (1.4) ***	**0.027**
Gynoid Fat, kg	7.8 (3.0)	6.8 (2.9) ***	7.9 (2.7)	7.4 (2.5) **	0.068
Visceral Fat, kg	1.6 (1.3)	1.4 (1.0) ***	1.8 (0.9)	1.6 (0.9)	**0.019**
**Region Lean Mass—DXA**					
Total lean, kg	50.6 (11.4)	50.1 (1.2)	52.7 (7.2)	52.4 (7.7)	0.997
Trunk lean, kg	23.7 (5.5)	23.3 (5.7) *	24.0 (3.1)	23.8 (3.3)	0.804
Android lean, kg	3.8 (1.0)	3.7 (1.0) **	3.8 (0.6)	3.8 (0.6)	0.542
Gynoid lean, kg	8.1 (1.9)	7.8 (1.9) **	8.1 (1.1)	8.1(1.2)	0.250
Appendicular lean, kg	23.7 (5.8)	23.6 (6.4)	25.3 (4.3)	25.3 (4.5	0.996
**Serum Analytes**					
Glucose, mg/dl	109.4 (39.6)	103.4 (12.6)	107.7 (33.5)	116.7 (59.4)	0.2529
Insulin, µU/mL	14.0 (9.1)	12.3(8.2)	18.0 (11.1)	21.5 (18.2)	0.1230
HOMA-IR	4.0 (3.4)	3.2 (2.4) ***	5.4 (5.7)	6.3 (5.9) *	0.0627
Triglycerides, mg/dl	121.0 (69.1)	96.0 (61.1) *	134.9 (75.3)	132.3 (83.7)	0.0875
HDL, mg/dl	66.1 (16.6)	65.3 (16.2)	58.1(16.2)	58.5 (12.5)	0.9360
LDL, mg/dl	113.0 (26.6)	113.5 (30.5)	101.3 (36.3)	104.4 (30.1)	0.3278
Total cholesterol, mg/dl	203.3 (31.1)	198.0 (35.8)	186.4 (35.8)	184.3 (30.8)	0.6262

Data given as mean (SD); DXA—Dual X-ray-Absorptiometry; * *p* < 0.05; ** *p* < 0.01; *** *p*< 0.001 for paired-t test comparing baseline and follow-up. *p* value for ANCOVA comparison of change variable between LCHF and LF groups, controlling for age, sex, race, and baseline measures. Bold *p*-values indicate significant.

**Table 3:** Comparison of change between baseline and follow up by insulin sensitivity and diet group with pairwise contrast within and between diet group.

	LCHF Diet (*n* = 32)		LF Diet (*n*=18)		Within Diet Contrasts		Between Diet Contrasts	
					LCHF Diet	LF Diet	IR	IS
	Insulin Resistant (*n* = 17)	Insulin Sensitive (*n* = 15)	Insulin Resistant (*n* = 11)	Insulin Sensitive (*n* = 7)	IR vs. IS	IR vs IS	LCHF vs. LF	LCHF vs. LF
**Demographics**					***p*-Value**	***p*-Value**	***p*-Value**	***p*-Value**
Weight, kg	−6.3 (5.3)	−6.0 (5.2)	−2.5 (4.6)	−4.2 (4.4)	0.7730	0.4432	0.0761	0.3207
BMI, kg/m^2^	−2.2 (1.9)	−2.2 (2.0)	−0.9 (1.8)	−1.4 (1.4)	0.7479	0.5400	0.0896	0.2694
Fat, %	−2.7 (3.0)	−2.8 (2.7)	−1.4 (1.1)	−1.6 (1.9)	0.9672	0.8715	0.1682	0.2785
REE, kcal/day	−46.2 (223.4)	3.9 (134.3)	−92.4 (14.6)	−38.2 (140)	0.7855	0.5209	0.2949	0.6271
**Serum Analytes**								
Glucose, mg/dL	−17.3 (45.3)	3.9 (7.6)	13.6 (39.5)	1.6 (12.3)	0.1702	0.3926	0.1093	0.7917
Insulin, µU/mL	−6.4 (8.8)	2.5 (7.5)	2.5 (19.9)	5 (10.9)	0.1016	0.7273	0.0827	0.7624
HOMA−IR	−2.4 (3.8)	0.7 (1.9)	0.8 (6.1)	1.2 (2.5)	0.8911	0.8608	0.0267 *	0.0895
Triglycerides, mg/dL	−42.6 (79.2)	−9.6 (27.9)	−5.6 (64.9)	2.1 (21.8)	0.2057	0.8310	0.0614	0.7537
HDL, mg/dL	−2.4 (8.6)	0.6 (10.2)	3.6 (8.1)	−4.7 (9.4)	0.0448 *	0.5996	0.3598	0.1342
LDL, mg/dL	1.5 (35.4)	−0.4 (22.2)	−0.3 (10.5)	−3.4 (26.0)	0.5551	0.7674	0.9664	0.3967
Total cholesterol, mg/dl	−9.5 (34.5)	−1.7 (25.9)	−1.5 (15.3)	−7.7 (21.8)	0.1132	0.5854	0.3945	0.2539
**Region Fat Mass—DXA**								
Total Fat, kg	−6.0 (3.4)	−4.8 (3.8)	−2.7 (3.1)	−3.4 (3.5)	0.7579	0.6532	0.1341	0.3413
Appendicular Fat, kg	−3 (4.0)	−1.8 (1.4)	−0.7 (1.0)	−1.5 (1.8)	0.1523	0.5187	0.0298 *	0.9288
Trunk Fat, kg	−4.1 (2.4)	−2.9 (2.5)	−1.9 (2.7)	−1.8 (1.8)	0.5034	0.9746	0.0844	0.0844
Android Fat, kg	−0.9 (0.5)	−0.6 (0.5)	−0.4 (0.4)	−0.4 (0.3)	0.3810	0.9198	0.0367 *	0.2895
Gynoid Fat, kg	−1 (0.7)	−0.9 (0.7)	−0.4 (0.7)	−0.8 (0.6)	0.8396	0.2321	0.0395 *	0.6548
Visceral Fat, kg	−0.4 (0.4)	−0.2 (0.2)	0.0 (0.3)	−0.1 (0.2)	0.1606	0.6264	0.0182 *	0.6133
**Region Lean Mass—DXA**								
Total lean, kg	−0.3 (2.4)	−0.6 (2.0)	0.2 (1.6)	−1 (2.5)	0.7220	0.2214	0.5537	0.2876
Trunk lean, kg	0.2 (1.3)	−0.3 (1.3)	0.2 (1.6)	−0.3 (1.3)	0.6762	0.4284	0.9850	0.6177
Android lean, kg	−0.5 (1.2)	−0.3 (1.0)	0 (1.1)	−0.6 (1.3)	0.2428	0.2961	0.2210	0.3196
Gynoid lean, kg	−0.2 (0.3)	−0.1 (0.2)	0 (0.1)	−0.1 (0.3)	0.2888	0.4527	0.0558	0.9607
Appendicular lean, kg	−0.2 (0.4)	−0.2 (0.3)	−0.1 (0.4)	−0.1 (0.3)	0.7326	0.7984	0.3111	0.7242

Data given as means (s.d.). IR-Insulin Resistant. IS-Insulin Sensitive. DXA-Dual X-ray-Absorptiometry. Paired contrasts adjusted for sex (M/W) and race (EA/AA). * *p* < 0.05.

## Data Availability

Due to the nature of this research, participants of this study did not agree for their data to be shared publicly, so supporting data is not available.

## Supplementary Materials

The following are available online at https://www.mdpi.com/2072-6643/13/2/475/s1, Table S1: Self-Reported Medical History of Study Participants. Table S2: Self-Reported Medication Use of Study Participants.
