# Supplementary material for: Greater Loss of Central Adiposity from Low-Carbohydrate versus Low-Fat Diet in Middle-Aged Adults with Overweight and Obesity
Source: Nutrients. 2021 Jan 31;13(2):475. doi: 10.3390/nu13020475 (PMC7911139; doi:10.3390/nu13020475)
Supplement: Supplementary file 1 [file nutrients-13-00475-s001.pdf]

nutrients MDPI

Article

Greater Loss of Central Adiposity from Low-Carbohydrate versus Low-Fat Diet in Middle-Aged Adults with Overweight and Obesity

Valene Garr Barry; Mariah Stewart; Taraneh Soleymani; Renee A. Desmond; Amy M. Goss and Barbara A. Gower *

Table S1. Self-reported medication use of study participants (N=50).

Medication Type	% of Sample
Anti-hypertensive	25
Thyroid	5
Pain medications	10
Statin	10
Blood Thinners	5
Proton Pump Inhibitors	15
Anti-diabetes	15
Allergy medications	15
Anti-depressants	5
Dyslipidemia	5
Insomnia	5
Diuretics	10
Anti-viral	5
NSAIDs	5
Vitamin supplements	10
Anti-psychotics	10
Hormone Replacement Therapy	10

Table S2. Self-reported medical history of study participants (N=50).

Medical History	% of Sample
High Blood Pressure	42
Thyroid Disease	8
Arthritis	20
Murmur	12
Heart Attack	2
Reflux/Heartburn	22
Diabetes	8
Asthma	16
Depression	28
Cataracts	8
High Cholesterol	28
Anxiety	20
Epilepsy	2
Colitis	4
Sleep Apnea	14
Cataracts	2
Pancreatitis	2
Pulmonary Embolism	4
Kidney Stones	8
Cancer	4
Hepatitis	2
